# Biological Evaluation of Silver-Treated Silk Fibroin Scaffolds for Application as Antibacterial and Regenerative Wound Dressings

**DOI:** 10.3390/nano15120919

**Published:** 2025-06-13

**Authors:** Federica Paladini, Carmen Lanzillotti, Angelica Panico, Mauro Pollini

**Affiliations:** 1 Department of Experimental Medicine, University of Salento, Via Monteroni, 73100 Lecce, Italy; 2 Caresilk S.r.l.s., Via Monteroni c/o Technological District DHITECH, 73100 Lecce, Italy; carmen.lanzillotti@caresilk.it (C.L.); angelica.panico@caresilk.it (A.P.)

**Keywords:** fibroin, silver, wound healing, biocompatibility, regenerative properties, antimicrobial activity

## Abstract

Chronic wounds represent a major clinical challenge due to their prolonged healing process and susceptibility to bacterial colonization, particularly by biofilm-forming bacteria. To address these issues, in this work, silver-treated silk fibroin scaffolds were developed and tested as multifunctional wound dressings, combining antimicrobial and regenerative properties. Silk fibroin, a natural protein derived from *Bombyx mori* cocoons, is widely recognized for its biocompatibility and suitability for tissue engineering. In this study, porous silk fibroin scaffolds were functionalized with silver nanoparticles through a photo-reduction process and were comprehensively tested for their cytocompatibility and wound healing potential. The excellent antibacterial activity of the silver-treated scaffolds was demonstrated against *Escherichia coli* and antibiotic-resistant *Pseudomonas aeruginosa*, as was extensively reported in a previous work. Biological assays were performed using 3T3 fibroblasts cultured on both untreated and silver-treated silk fibroin scaffolds. Biocompatibility assays, such as MTT, Live/Dead, and cytoskeleton analyses, demonstrated biocompatibility in both scaffold types, comparable to the control. Wound healing potential was assessed using in vitro scratch assays, revealing full wound closure (100%) after 24 h in cells cultured with untreated and silver-treated silk fibroin scaffolds, in contrast to 78.5% closure in the control. Notably, silver-treated scaffolds exhibited enhanced fibroblast repopulation within the wound gap, suggesting a synergistic effect of silver and fibroin in promoting tissue regeneration. These findings demonstrate that silver-treated silk fibroin scaffolds possess both anti-microbial and regenerative properties, making them promising candidates for chronic wound management applications.

## 1. Introduction

Chronic wounds represent a significant and persistent global healthcare burden, affecting approximately 1–2% of the population in developed countries [1]. Their prevalence is steadily increasing due to the aging of the global population and the rising incidence of underlying comorbidities such as diabetes mellitus, obesity, peripheral vascular disease, and venous insufficiency [2,3]. These wounds, which include diabetic foot ulcers, pressure ulcers, and venous leg ulcers, are notoriously difficult to treat and often become recalcitrant, leading to prolonged patient suffering, frequent hospitalizations, and a considerable economic impact on healthcare systems. Unlike acute wounds, which follow a predictable healing process, chronic wounds are characterized by the failure to progress through the normal stages of wound healing, namely haemostasias, inflammation, proliferation, and remodeling, in an orderly and timely manner [4,5,6]. Most chronic wounds typically remain in the inflammatory phase for extended periods, leading to significant morbidity, reduced quality of life, and substantial healthcare costs [7,8,9]. The pathophysiology of chronic wounds is complex and involves multiple interconnected factors that create a self-perpetuating cycle of delayed healing, such as local tissue hypoxia, bacterial colonization, excessive inflammatory response, and impaired cellular function [5,10,11]. Bacterial colonization represents a critical challenge in chronic wound management, with biofilms playing a particularly detrimental role in healing failure [12]. Biofilms are sophisticated three-dimensional structures composed of bacterial communities encased within a self-produced extracellular polymeric matrix consisting of polysaccharides, proteins, lipids, and extracellular DNA [13,14,15]. This protective matrix shields bacteria from the host’s immune response, antimicrobial agents, and environmental stresses, creating a persistent source of infection and inflammation [16,17]. In chronic wounds, biofilm formation occurs through a well-characterized process, including (i) initial bacterial adhesion to the wound surface, (ii) microcolony formation and early matrix production, (iii) biofilm maturation with complex architecture development, and (iv) the dispersal of planktonic bacteria to colonize new sites [13,18]. The moist, nutrient-rich environment of chronic wounds, combined with compromised host defenses, provides ideal conditions for this process. The clinical impact of biofilms is profound, with studies indicating that ~80% of chronic wounds contain biofilm-forming bacteria compared to only 6% of acute wounds [19,20]. Common biofilm-forming pathogens in chronic wounds include *Staphylococcus aureus*, *Pseudomonas aeruginosa*, *Streptococcus species*, and *Enterococcus species*, often existing in polymicrobial communities, which exhibit enhanced resistance to treatment [21]. These biofilms prolong the inflammatory response, delay healing, and significantly increase the risk of infection and systemic complications, including amputation and even mortality [22,23,24,25]. Despite advances in wound care technology, conventional wound dressings demonstrate significant limitations in addressing the complex pathophysiology of chronic wounds [26]. Traditional passive dressings, including gauze, cotton, and basic adhesive bandages, only provide physical protection and moisture absorption without addressing the underlying biological dysfunction [27,28]. The limitations of current wound care technologies highlight the need for innovative multifunctional approaches that can simultaneously address multiple aspects of chronic wound pathophysiology. Ideal next-generation wound dressings should combine antimicrobial activity, biofilm disruption, cellular support, and regenerative properties in a single platform. Silver, a well-known antimicrobial agent with its effectiveness against more than 600 microorganisms being recognized, including against antibiotic-resistant bacteria, represents a valuable opportunity to develop antimicrobial wound dressings [14,15,29]. In the form of nanoparticles, silver in particular has demonstrated excellent microbiological properties due its the high volume to surface ratio [18], along with its possibility of being incorporated in multiple categories of products [20,21,30]. The antimicrobial mechanism of silver develops through multiple pathways, involving the disruption of bacterial cell wall integrity through electrostatic interactions with negatively charged bacterial surfaces, penetration into bacterial cells, and binding to sulfur-containing proteins and DNA. Other mechanisms determine the generation of reactive oxygen species that lead to oxidative stress and the interference with bacterial enzyme systems essential for cellular respiration [18,31].

Silk fibroin, a natural protein derived from silk cocoons, is an extremely promising material for tissue engineering due to its exceptional biocompatibility and its capability of supporting cell adhesion, proliferation, and migration [32,33,34]. Silk fibroin can be processed into a wide range of formats, including sponges, films, nanofibers, and hydrogels, making it a highly adaptable material for biomedical applications [35,36].

In this study, silk fibroin scaffolds, properly developed for wound healing applications, were functionalized with silver nanocoatings, aiming to combine the regenerative properties of this protein with the antimicrobial activity of silver [28,37,38]. A comprehensive biological evaluation of the prototypes was performed in order to investigate any potential cytotoxic effect of the treatment on the fibroin scaffold and also to analyze the potential wound healing properties for application in critical wound management.

## 2. Materials and Methods

Silk fibroin scaffolds, previously characterized by their mechanical and antibacterial performance [28], were supplied by Caresilk S.r.l.s. (Lecce, Italy). The scaffolds were manufactured using a proprietary method specifically designed to create a sponge-like structure with enhanced porosity (average pore size ~300 μm), improved flexibility, and extended degradation time compared to conventional freeze-dried fibroin scaffolds. The scaffolds demonstrated a swelling ratio of approximately 1200% and maintained structural integrity for over 7 days in physiological conditions.

### 2.1. Silver Deposition Treatment

Silver coatings were deposited onto the surface of silk fibroin scaffolds using a silver deposition technique based on the in situ photochemical deposition of silver nanoparticles [28]. Briefly, the silver treatment consists of the preparation of the silver solution (0.1% wt/wt silver nitrate, 5% wt/wt methanol, 94.9% wt/wt deionized water), its deposition by spray coating it on the surface of the material, and the following exposure to ultraviolet light (500 W, λ = 365 nm) for 10 min per side. This method promotes the conversion of the silver precursor into metallic silver and enables the direct synthesis of silver nanoparticles on the substrate. Chemicals and reagents, such as silver nitrate, methanol, and deionized water, were purchased from Sigma Aldrich (St. Louis, MO, USA).

### 2.2. Biocompatibility Evaluation

The biocompatibility of both untreated and silver-treated silk fibroin scaffolds was evaluated using NIH-3T3 murine fibroblasts at passage 3. Cells were expanded in Dulbecco’s Modified Eagle Medium (DMEM; Sigma Aldrich), supplemented with 10% fetal bovine serum (FBS), 1% antibiotics (100 U/mL penicillin, 100 mg/mL streptomycin), and 2 mM L-glutamine, and were maintained in a humified incubator (Heracell, Thermo Scientific, Waltham, MA, USA) at 37 °C with 5% CO_2_. The medium was changed every three days. 3T3 fibroblasts were seeded at a concentration of 1.5 × 10^4^ cells per well and were grown in contact with untreated (UT-SF) and treated (T-SF) silk fibroin scaffolds, whereas a concentration of 5 × 10^3^ cells was grown on tissue culture polystyrene (TCPS) as the control.

### 2.3. MTT Assay

An MTT assay [3-(4,5-dimethylthiazol-2-yl)-2,5-diphenyltetrazolium bromide; Sigma Aldrich] was performed to assess the cell viability of the UT-SF and T-SF silk fibroin scaffolds. 3T3 fibroblasts were cultured in direct contact with the samples and with TCPS as the control. The MTT assay was performed in triplicate for each sample type at 3, 5, and 7 days of incubation, and three independent experiments were carried out. Specifically, the MTT solution (5 mg/mL in PBS) was added to fresh medium in each well to reach a final concentration of 0.5 mg/mL. Plates were incubated at 37 °C for 3 h, and the resulting formazan crystals were dissolved using DMSO. The absorbance was measured at 540 nm using a V-1200 spectrophotometer (Avantor, Inc., VWR, Radnor Township, PA, USA) [39].

### 2.4. Live/Dead Assay

A Live/Dead assay was carried out on 3T3 fibroblasts in contact with the UT-SF and T-SF silk fibroin scaffolds and with TCPS as the control to further evaluate the scaffolds’ biocompatibility. The assay was performed at 5 and 7 days in three independent experiments. Cells, grown directly on a coverslip, were then incubated for 15 min at 37 °C with a staining solution containing 2 μmol/L calcein-AM (acetomethoxy, a derivative of calcein) and 2 μmol/L propidiumiodide in PBS. Afterwards, an analysis of live and dead cells was carried out using a fluorescence microscope (Axio Vert A1, Zeiss, Oberkochen, Germany) at 20× magnification and AxioVision software (Zeiss ZEN 3.11) [40].

### 2.5. Cytoskeleton Architecture Analysis

The cytoskeleton architecture of the 3T3 fibroblast cultured on the UT-SF and T-SF scaffolds and on TCPS as the control was analyzed on 5 and 7 days in three independent experiments. Cells were fixed in 4% paraformaldehyde for 20 min at room temperature. Subsequently, cells were permeabilized with 0.5% of Titon-X100 solution for 10 min following a PBS wash. Cytoskeleton filaments were stained using tetramethylrhodamine isothiocyanate (TRITC)-conjugated phalloidin (Sigma Aldrich), whereas the nuclei were stained with 0.5 mg/mL 4′,6-diamidino-2-phenylindole (DAPI; Invitrogen, Waltham, MA, USA). Fluorescence microscopy was performed at 20× magnification using a microscope (Axio Vert A1, Zeiss), and image analyses were performed using AxioVision software (Zeiss ZEN 3.11) [40].

### 2.6. In Vitro Scratch Assay

The scratch assay, consisting of an injury to the cell monolayer, was carried out on 3T3 fibroblast grown in contact with the UT-SF and T-SF scaffolds and with TCPS as the control in order to assess wound-healing capability and, thus, the regenerative property of silk fibroin scaffolds. 3T3 fibroblasts were seeded in 24-well plates at a density of 1 × 10^5^ cells/mL and cultured until confluence. A linear scratch (~1 mm wide) was made across the monolayer using a sterile pipette tip. After washing with PBS to remove debris, the cells were maintained in culture with scaffolds. Wound closure was monitored by capturing images at 0 and 24 h using an optical microscope (Axio Vert A1, Zeiss). Analyses of the digital images were performed using ImageJ software 1.53e (National Institutes of Health Bethesda), and the results are expressed as the average percentage of wound closure (%) up to Time 1. Particularly, the wound closure rate was calculated using the following equation:

(1) WC (%) = {[(WA_T0_) − (WA_T1_)/(WA_T0_)] × 100}

where:

WC: wound closure;
WA_T0_: wound area at Time 0;
WA_T1_: wound area at Time 1.

as reported by Yewseok K. Suh et al. [41]. Two independent experiments were conducted.

### 2.7. Statistical Analysis

Statistical experimental analyses were carried out using the GraphPad Prism 8.0.1 software. Two-way ANOVA and multiple comparison tests were used to analyze the statistics of the MTT assay and wound closure rate results.

*p*-value < 0.05 was considered significant [42].

## 3. Results

### 3.1. Biocompatibility of Untreated and Silver-Treated Silk-Fibroin Scaffolds

The MTT assay was carried out to analyze the effect of UT-SF and T-SF scaffolds on 3T3 cell viability. The results show an increase of 540 nm absorbance, corresponding to an increase in cell number and of 3T3 grown on UT-SF and T-SF scaffolds, as well as on the control, up to day 7 [43].

**Figure 1:**
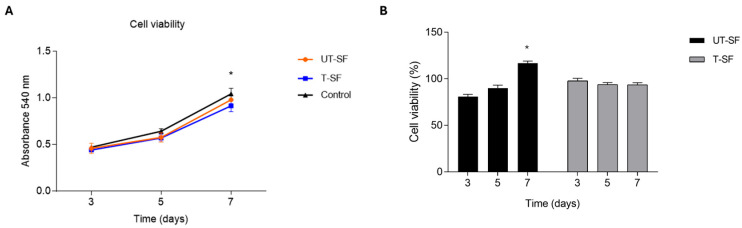
(**A**) Cell viability of 3T3 grown in contact with UT-SF and T-SF scaffolds and on the control at 3, 5, and 7 days using an MTT assay. Graph shows increasing absorbance (540 nm) from day 3 to day 7 in each experimental group. Cell viability significant increases from day 3 to 7 (*p* < 0.05), as well as from day 5 to 7 (*p* < 0.05). No significant difference is present among the experimental groups at each time point (*p* > 0.05). (* *p* < 0.05). (**B**) Percentage of cell viability. 3T3 cells were grown in contact with UT-SF and T-SF scaffolds at 3, 5, and 7 days, normalized to the control group. Percentage of cell viability of T-SF scaffold significant increased from day 3 to 7 (* *p* < 0.05), as well as from day 5 to 7 (*p* < 0.05).

There is significant difference in increasing cell number between day 3 and 7 (*p* < 0.05), as well as day 5 and 7 (*p* < 0.05), whereas there is no significant difference among the experimental groups at each time point (*p* > 0.05). The results confirm that both the UT-SF and T-SF scaffolds did not cause cytotoxicity and allow for 3T3 cell growth (Figure 1A). In addition, the percentage of cell viability at each time point was calculated by normalizing the absorbance of each experimental group to that of the control group. The percentage of cell viability of UT-SF was 81%, 90%, and 116% at 3, 5, and 7 days, respectively, whereas the percentage of cell viability of T-SF was 98%, 94%, and 94% at 3, 5, and 7 days, respectively (Figure 1B). For the UT-SF scaffold, the statistical analyses of cell viability percentages were consistent with the absorbance data, both showing a significant increase in cell viability between day 3 and day 7 (*p* < 0.05), as well as between day 5 and day 7 (*p* < 0.05). In contrast, for the T-SF scaffold, statistical analyses of viability percentages did not reveal any significant changes over time, unlike the absorbance data. Despite the lack of statistically significant variation, the cell viability values remained above the cytotoxicity threshold defined by ISO 10993-5:2009[44], which considers a reduction in cell viability higher than 30% to be indicative of cytotoxicity [45]. Overall, these results confirm the biocompatibility of both the UT-SF and T-SF scaffolds.

**Figure 2:**
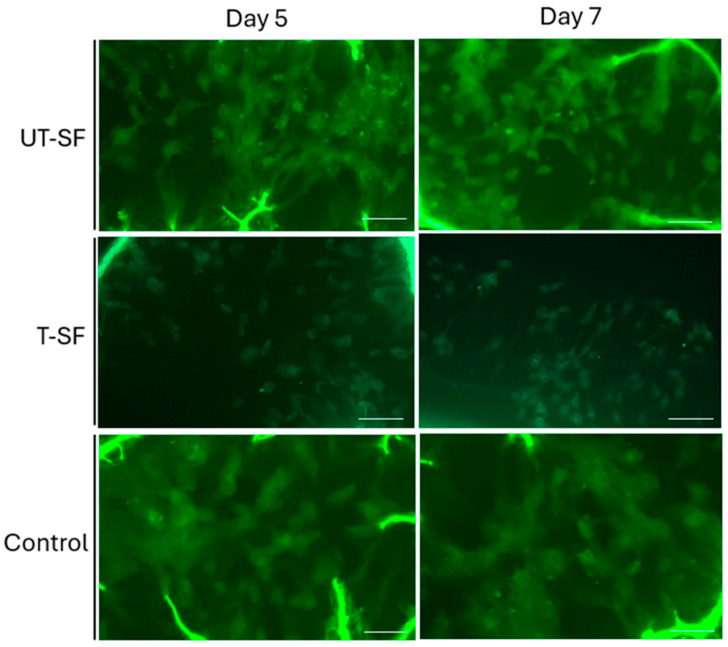
Cell viability analysis of 3T3 fibroblasts using Live/Dead assay. Images show live cells grown in contact with UT-SF and T-SF scaffolds and on the control at 5 and 7 days. Dead cells at each time point were not detected. Scale bar: 50 μm.

The in vitro biocompatibility of the two types of scaffolds was also investigated using Live/Dead fluorescent assay at days 5 and 7. The green and red fluorescent dyes were used to stain live and dead cells, respectively. At each time point, digital images showed the presence of live 3T3 cells grown in contact with UT-SF and T-SF scaffolds, comparable to the control (Figure 2). Dead cells were not observed , confirming the cytocompatibility of both the UT-SF and T-SF silk fibroin scaffolds.

**Figure 3:**
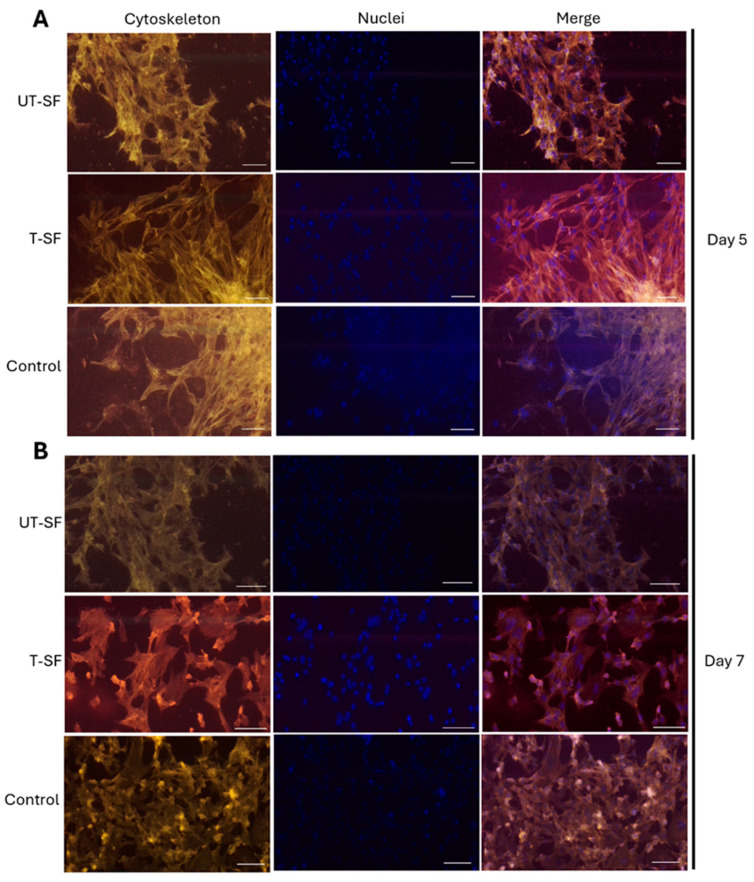
(**A**) Cytoskeleton analysis of 3T3 grown in contact with UT-SF and T-SF scaffolds and on the control at day 5. The structure of cytoskeleton does not show an alteration in 3T3 grown on UT-SF and T-SF compared to the control. (**B**) Cytoskeleton analysis of 3T3 grown in contact with UT-SF and T-SF scaffolds and on the control at day 7. The structure of cytoskeleton does not show an alteration in 3T3 grown on UT-SF and T-SF compared to the control. Magnification ×20; scale bar: 50 μm. Cellular nuclei were stained with 0.5 mg/mL 4′,6-diamidino-2-phenylindole (DAPI).

The cytoskeleton organization of 3T3 fibroblasts cultured in contact with UT-SF and T-SF scaffolds and on TCPS used as the control were evaluated using phalloidin-TRITC staining on day 5 (Figure 3A) and day 7 (Figure 3B). A well-organized cytoskeleton structure was observed in cells grown in contact with the UT-SF and T-SF silk fibroin scaffolds, as well as on the control, at days 5 and 7. Digital microscopy images at 20× magnifications (Figure 3) revealed unaltered actin filaments, further indicating the cytocompatibility of the UT-SF and T-SF silk fibroin scaffolds that did not affect cytoskeleton architecture up to day 7.

### 3.2. Wound Healing Properties of Untreated and Silver-Treated Silk-Fibroin Scaffolds

**Figure 4:**
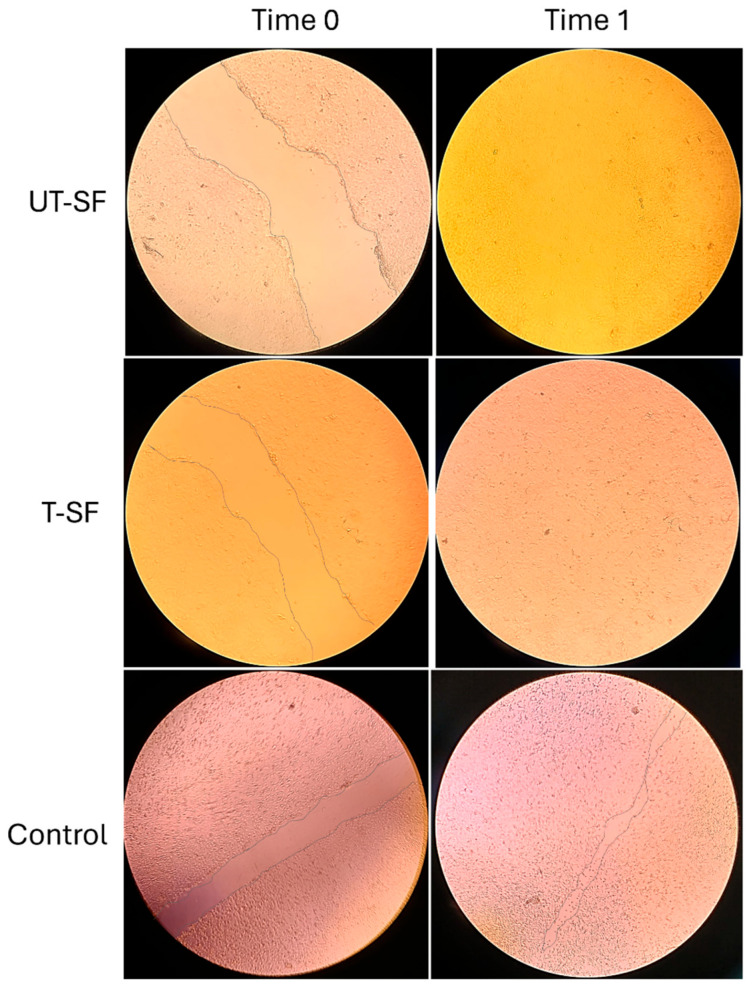
Wound healing analysis through an in vitro scratch assay on 3T3 grown in contact with UT-SF and T-SF scaffolds and on control, at Time 0 and Time 1 of analysis. At Time 1, the wound closure is completed in UT-SF and T-SF groups, differently from the control.

**Figure 5:**
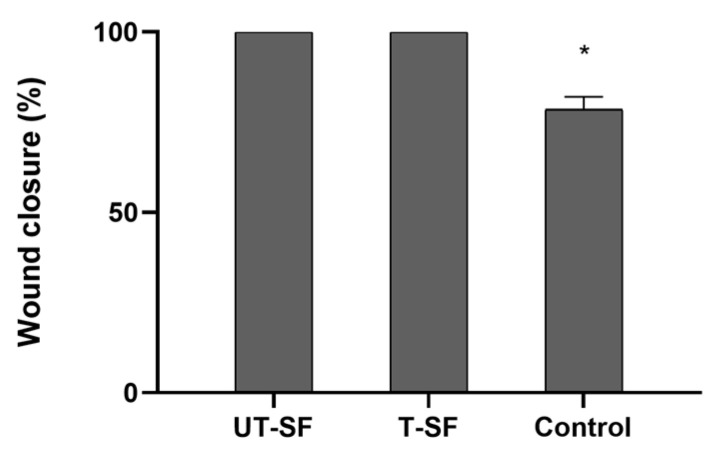
Quantification of wound gap distance (%) using ImageJ software at Time 1. Graph reports mean of measurements of the two independent experiments. At Time 0, the mean of the wound closure rate is 0% in all experimental groups (not showed in the graph). At Time 1, the mean of the wound closure rate is 100% in 3T3 grown in contact with UT-SF and T-SF scaffolds, whereas it is 78.5% in the control group (*p* < 0.05). (* *p* < 0.05).

The wound healing properties of UT-SF and T-SF silk fibroin scaffolds were assessed on 3T3 fibroblasts grown in contact with samples and with the control. Specifically, wound healing was investigated in vitro using a scratch assay, conducted at the initial time point (Time 0) and after 24 h of incubation (Time 1). Microscopy images show the scratched fibroblast monolayer of UT-SF, T-SF, and control samples at Time 0 and Time 1 (Figure 4), illustrating the wound closure of the three experimental groups. At Time 1, the wound closure rate is completed (100%) in UT-SF and T-SF, whereas in the control sample, the wound closure rate is 78.5% (Figure 5). In addition, digital images show a stronger repopulation of fibroblasts in the T-SF compared to the UT-SF samples (Figure 5). The results reveal that the presence of silk fibroin enhanced the wound closure and that the presence of silver further improved the wound healing capacity of the scaffold. These findings demonstrate the regenerative properties of silk fibroin scaffolds and their synergistic activity with silver.

A summary table is provided below to offer a clearer overview of the experimental outcomes. Table 1 reports the percentage of cell viability at days 3, 5, and 7, as well as the wound closure rate at time 1. Additionally, key observations related to the bioactivity of the scaffolds are included.

## 4. Discussion

Current approaches for chronic wound management include the key elements collected in the acronym “TIME”, which indicates Tissue management, Infection/inflammation control, Moisture balance, and Edge of wound [46,47]. Indeed, the maintenance of a moist environment while removing the exudate excess, protection of the wound bed from contamination, and good thermal and gas exchange are important features that an ideal wound dressing should provide to contribute to the wound healing process [48]. Advanced wound dressings developed using biomaterial-based tissue engineering strategies have emerged as promising alternatives for chronic wound management [49]. Being amongst the most appealing biomaterials for tissue engineering, silk fibroin derived from *Bombyx mori* has garnered significant attention due to its exceptional combination of biocompatibility, mechanical durability, and versatility in fabrication [50,51]. From a technological point of view, fibroin offers great advantages that allow for it to be processed into diverse architecture, such as films, sponges, hydrogels, electrospun nanofibers, and composite scaffolds [52], thus enabling the development of fibroin-based wound healing platforms and the incorporation of bioactive molecules such as growth factors, antimicrobial peptides, and anti-inflammatory agents [33,53]. This study aims at providing advances beyond the current state of the art in multifunctional wound dressing development and to develop a biomaterial with enhanced antimicrobial and regenerative performances. For this purpose, the approach of this work is based on the combination between the advantages related to the photochemical silver deposition technology used in previous works on different substrates with properly designed engineered silk fibroin scaffolds. Unlike conventional approaches based on simply mixed silver nanoparticles with biomaterials or post-synthesis incorporation methods [52], the adopted photochemical in situ deposition technique creates silver nanostructures directly on the fibroin matrix. This approach offers several advantages over existing methods, such as the superior adhesion of the silver coating, the preservation of the protein structural integrity, and homogeneous surface coverage without bulk material modification [28]. Moreover, in addition to the well-known properties of silver and fibroin, this work aims at evaluating their synergistic effects in wound healing applications for future consideration in clinical practice. For this purpose, as a first stage of the research, the effect of the silver treatment on the features of the scaffold has been investigated in a previous work in terms of its chemical–physical and antibacterial properties. The results obtained in the previous research demonstrated that silver treatment does not affect significantly the properties of the scaffold, while providing it with antibacterial properties against *Escherichia coli* and antibiotic-resistant *Pseudomonas aeruginosa* as representative bacterial strains in critical wound infections [28]. Microbiological characterization was performed using both qualitative and quantitative assays at different degradation time points. The results of the agar diffusion test indicate the good antibacterial activity of the silver-treated samples against both bacterial strains, with an inhibition zone larger than 1 mm appearing even after one week. Quantitative tests confirmed these findings and enabled the evaluation of antibacterial efficacy in terms of bacterial proliferation reduction. Compared to the sample at t = 0, which showed a 95% reduction for *Escherichia coli* and 92% for antibiotic-resistant *Pseudomonas aeruginosa*, subsequent time points confirmed that, despite the very low amount of silver used, the treatment remained effective in providing antibacterial activity throughout the entire degradation experiment [28]. The present work focuses on the biological evaluation of the silver-treated silk fibroin scaffold in comparison to the control sample in order to investigate the potential cytotoxic effects of silver in terms cell viability and proliferation. Moreover, the regenerative properties of the device were investigated in vitro using a scratch assay, aiming to assess the potential impact of the silver coating on the intrinsic regenerative capacity of the fibroin scaffold. The biological tests evidenced the good biocompatibility of both the untreated and the silver-treated samples, confirming the results obtained in previous works on different medical devices when using the same deposition technology [50], where cell functions were not affected by the presence of the silver coating. Interestingly, along with the absence of cytotoxic effects, even higher bioactivity was observed in the treated scaffold in terms of wound closure, suggesting a synergistic action between silver and fibroin that can further accelerate the wound healing process. This synergistic effect may be attributed to silver’s ability to modulate inflammatory responses while promoting angiogenesis [54]. The enhanced fibroblast migration observed in silver-treated scaffolds could be related to the upregulation of wound healing-associated genes such as VEGF and TGF-β1. In particular VEGF, which promotes both angiogenesis and fibroblast chemotaxis, could be directly related to the increased cell density observed in wound gap analysis. TGF-β1 is a regulator of fibroblast proliferation, collagen synthesis, and epithelial–mesenchymal transition. Silver-induced TGF-β1 modulation could account for the accelerated wound closure kinetics observed in the scratch assays [55,56,57,58].

These findings align with the growing academic interest in multifunctional wound dressings that integrate different properties, such as antimicrobial activity, biocompatibility, and regenerative potential, into a single platform.

Similar multifunctional strategies have been explored in the recent literature. For example, Castillo Ortega et al. developed Aloe Vera mucilage-loaded gelatin electrospun fibers, demonstrating promising bioactivity and structural support for tissue engineering applications [59]. Other recent studies have demonstrated the benefits of combining silver with biopolymers such as chitosan or collagen to enhance wound healing outcomes through antimicrobial, anti-inflammatory, and pro-angiogenic mechanisms [60,61]. These findings further support the rationale behind this work, which also offers advantages related to an innovative and scalable fabrication process, along with high antimicrobial efficacy, including activity against antibiotic-resistant strains, and significant regenerative potential.

## 5. Conclusions

The increased need for the definition of effective strategies in wound management has stimulated the development of novel wound dressing biomaterials for preventing infection and promoting tissue regeneration. In this work, silver-treated silk fibroin scaffolds have been developed, and their biological potential has been comprehensively evaluated. Key findings from this study include considerations about biocompatibility, enhanced wound healing, synergistic effects, and structural integrity. In particular, both untreated and silver-treated silk fibroin scaffolds demonstrated excellent biocompatibility, with cell viability exceeding 80% across all time points tested, confirming the maintenance of cellular function after silver treatment. Moreover, the silver-treated scaffolds achieved 100% wound closure within 24 h compared to 78.5% in controls, with significantly enhanced fibroblast repopulation in the wound area. The combination of silk fibroin and silver demonstrated synergistic activity, with silver not only providing antimicrobial properties, as demonstrated in a previous work, but also actively promoting wound healing processes. Cytoskeleton analysis revealed that silver treatment did not compromise cellular architecture or function, maintaining normal actin filament organization. The results suggest that the developed device represents a promising candidate for critical wound applications, providing simultaneous protection against infections and accelerating the wound healing process.

## Figures and Tables

**Table 1:** Summary table that includes the key findings related to the percentage of cell viability, wound closure rate, and observed bioactivity of UT-SF and T-SF samples.

Sample	Cell Viability (%—Day 3)	Cell Viability (%—Day 5)	Cell Viability (%—Day 7)	Wound Closure Rate (%—Time 1)	Observed Bioactivity
UT-SF	81	90	116	100	Biocompatible; regenerative
T-SF	98	94	94	100	Biocompatible; regenerative; synergistic effect of silver and fibroin

## Data Availability

The original contributions presented in this study are included in the article. Further inquiries can be directed to the corresponding author(s).

## Abbreviations

The following abbreviations are used in this manuscript:

DMEM	Dulbecco’s Modified Eagle Medium
FBS	Fetal Bovine Serum
UT-SF	Untreated silk fibroin scaffold
T-SF	Treated silk fibroin scaffold
TCPS	Tissue Culture Polystyrene
MTT	3-(4,5-dimethylthiazol-2-yl)-2,5-diphenyltetrazolium bromide
DMSO	Dimetilsolfossido
AM	Acetomethoxy
PBS	Phosphate-Buffered Saline
TRICT	Tetramethylrhodamine isothiocyanate
DAPI	4′,6-diamidino-2-phenylindole

## References

[1] Falcone M., De Angelis B., Pea F., Scalise A., Stefani S., Tasinato R., Zanetti O., Paola L.D. (2021). Challenges in the management of chronic wound infections. J. Glob. Antimicrob. Resist..

[2] Frykberg R.G., Banks J. (2015). Challenges in the Treatment of Chronic Wounds. Adv. Wound Care.

[3] Uberoi A., McCready-Vangi A., Grice E.A. (2024). The wound microbiota: Microbial mechanisms of impaired wound healing and infection. Nat. Rev. Microbiol..

[4] Makrantonaki E., Wlaschek M., Scharffetter-Kochanek K. (2017). Pathogenesis of wound healing disorders in the elderly. J. Dtsch. Dermatol. Ges. J. Ger. Soc. Dermatol..

[5] Guo S., Dipietro L.A. (2010). Factors affecting wound healing. J. Dent. Res..

[6] Wang S., Wu W.-Y., Yeo J.C.C., Soo X.Y.D., Thitsartarn W., Liu S., Tan B.H., Suwardi A., Li Z., Zhu Q. (2023). Responsive hydrogel dressings for intelligent wound management. BMEMat.

[7] Olsson M., Järbrink K., Divakar U., Bajpai R., Upton Z., Schmidtchen A., Car J. (2019). The humanistic and economic burden of chronic wounds: A systematic review. Wound Repair Regen. Off. Publ. Wound Health Soc. Eur. Tissue Repair Soc..

[8] Sen C.K. (2021). Human Wound and Its Burden: Updated 2020 Compendium of Estimates. Adv. Wound Care.

[9] Bowers S., Franco E. (2020). Chronic Wounds: Evaluation and Management. Am. Fam. Physician.

[10] Schilrreff P., Alexiev U. (2022). Chronic Inflammation in Non-Healing Skin Wounds and Promising Natural Bioactive Compounds Treatment. Int. J. Mol. Sci..

[11] Wallace H.A., Basehore B.M., Zito P.M. (2025). Wound Healing Phases. StatPearls.

[12] Goswami A.G., Basu S., Banerjee T., Shukla V.K. (2023). Biofilm and wound healing: From bench to bedside. Eur. J. Med. Res..

[13] Sharma S., Mohler J., Mahajan S.D., Schwartz S.A., Bruggemann L., Aalinkeel R. (2023). Microbial Biofilm: A Review on Formation, Infection, Antibiotic Resistance, Control Measures, and Innovative Treatment. Microorganisms.

[14] Lin Y.-H., Hsu W.-S., Chung W.-Y., Ko T.-H., Lin J.-H. (2016). Silver-based wound dressings reduce bacterial burden and promote wound healing. Int. Wound J..

[15] Cavanagh M.H., Burrell R.E., Nadworny P.L. (2010). Evaluating antimicrobial efficacy of new commercially available silver dressings. Int. Wound J..

[16] Sahoo K., Meshram S. (2024). Biofilm Formation in Chronic Infections: A Comprehensive Review of Pathogenesis, Clinical Implications, and Novel Therapeutic Approaches. Cureus.

[17] Cavallo I., Sivori F., Mastrofrancesco A., Abril E., Pontone M., Di Domenico E.G., Pimpinelli F. (2024). Bacterial Biofilm in Chronic Wounds and Possible Therapeutic Approaches. Biology.

[18] More P.R., Pandit S., Filippis A.D., Franci G., Mijakovic I., Galdiero M. (2023). Silver Nanoparticles: Bactericidal and Mechanistic Approach against Drug Resistant Pathogens. Microorganisms.

[19] Thaarup I.C., Iversen A.K.S., Lichtenberg M., Bjarnsholt T., Jakobsen T.H. (2022). Biofilm Survival Strategies in Chronic Wounds. Microorganisms.

[20] Picca R.A., Paladini F., Sportelli M.C., Pollini M., Giannossa L.C., Di Franco C., Panico A., Mangone A., Valentini A., Cioffi N. (2017). Combined Approach for the Development of Efficient and Safe Nanoantimicrobials: The Case of Nanosilver-Modified Polyurethane Foams. ACS Biomater. Sci. Eng..

[21] Sportelli M.C., Picca R.A., Paladini F., Mangone A., Giannossa L.C., Franco C.D., Gallo A.L., Valentini A., Sannino A., Pollini M. (2017). Spectroscopic Characterization and Nanosafety of Ag-Modified Antibacterial Leather and Leatherette. Nanomaterials.

[22] Metcalf D.G., Bowler P.G. (2013). Biofilm delays wound healing: A review of the evidence. Burns Trauma.

[23] Versey Z., da Cruz Nizer W.S., Russell E., Zigic S., DeZeeuw K.G., Marek J.E., Overhage J., Cassol E. (2021). Biofilm-Innate Immune Interface: Contribution to Chronic Wound Formation. Front. Immunol..

[24] Diban F., Di Lodovico S., Di Fermo P., D’Ercole S., D’Arcangelo S., Di Giulio M., Cellini L. (2023). Biofilms in Chronic Wound Infections: Innovative Antimicrobial Approaches Using the In Vitro Lubbock Chronic Wound Biofilm Model. Int. J. Mol. Sci..

[25] Di Domenico E.G., Farulla I., Prignano G., Gallo M.T., Vespaziani M., Cavallo I., Sperduti I., Pontone M., Bordignon V., Cilli L. (2017). Biofilm is a Major Virulence Determinant in Bacterial Colonization of Chronic Skin Ulcers Independently from the Multidrug Resistant Phenotype. Int. J. Mol. Sci..

[26] Ferraz M.P. (2025). Wound Dressing Materials: Bridging Material Science and Clinical Practice. Appl. Sci..

[27] Mihai M.M., Dima M.B., Dima B., Holban A.M. (2019). Nanomaterials for Wound Healing and Infection Control. Materials.

[28] Paladini F., Russo F., Masi A., Lanzillotti C., Sannino A., Pollini M. (2024). Silver-Treated Silk Fibroin Scaffolds for Prevention of Critical Wound Infections. Biomimetics.

[29] Dove A.S., Dzurny D.I., Dees W.R., Qin N., Rodriguez C.C.N., Alt L.A., Ellward G.L., Best J.A., Rudawski N.G., Fujii K. (2023). Silver nanoparticles enhance the efficacy of aminoglycosides against antibiotic-resistant bacteria. Front. Microbiol..

[30] Bruna T., Maldonado-Bravo F., Jara P., Caro N. (2021). Silver Nanoparticles and Their Antibacterial Applications. Int. J. Mol. Sci..

[31] Yin I.X., Zhang J., Zhao I.S., Mei M.L., Li Q., Chu C.H. (2020). The Antibacterial Mechanism of Silver Nanoparticles and Its Application in Dentistry. Int. J. Nanomed..

[32] Paladini F., Pollini M. (2022). Novel Approaches and Biomaterials for Bone Tissue Engineering: A Focus on Silk Fibroin. Materials.

[33] Lehmann T., Vaughn A.E., Seal S., Liechty K.W., Zgheib C. (2022). Silk Fibroin-Based Therapeutics for Impaired Wound Healing. Pharmaceutics.

[34] Melke J., Midha S., Ghosh S., Ito K., Hofmann S. (2016). Silk fibroin as biomaterial for bone tissue engineering. Acta Biomater..

[35] Li Z.-H., Ji S.-C., Wang Y.-Z., Shen X.-C., Liang H. (2013). Silk fibroin-based scaffolds for tissue engineering. Front. Mater. Sci..

[36] Soomherun N., Kriangsaksri R., Tanticharakunsiri W., Foongsawat N., Phoolcharoen W., Tawinwung S., Keeratihattayakorn S., Ratanavaraporn J. (2024). Silk fibroin-based hydrogel as injectable carrier for prolonged immunization of plant-based COVID-19 subunit vaccine. J. Drug Deliv. Sci. Technol..

[37] Cooper I.R., Pollini M., Paladini F. (2016). The potential of photo-deposited silver coatings on Foley catheters to prevent urinary tract infections. Mater. Sci. Eng. C Mater. Biol. Appl..

[38] Pollini M., Paladini F., Licciulli A., Maffezzoli A., Sannino A., Cioffi N., Rai M. (2012). Engineering Nanostructured Silver Coatings for Antimicrobial Applications. Nano-Antimicrobials: Progress and Prospects.

[39] Nirwana I., Munadziroh E., Yogiartono R.M., Thiyagu C., Ying C.S., Dinaryanti A. (2021). Cytotoxicity and proliferation evaluation on fibroblast after combining calcium hydroxide and ellagic acid. J. Adv. Pharm. Technol. Res..

[40] Lanzillotti C., Iaquinta M.R., De Pace R., Mosaico M., Patergnani S., Giorgi C., Tavoni M., Dapporto M., Sprio S., Tampieri A. (2024). Osteosarcoma cell death induced by innovative scaffolds doped with chemotherapeutics. J. Cell. Physiol..

[41] Suh Y.K., Robinson A., Zanghi N., Kratz A., Gustetic A., Crow M.M., Ritts T., Hankey W., Segarra V.A. (2022). Introducing Wound Healing Assays in the Undergraduate Biology Laboratory Using Ibidi Plates. J. Microbiol. Biol. Educ..

[42] Corazza M., Oton-Gonzalez L., Scuderi V., Rotondo J.C., Lanzillotti C., Di Mauro G., Tognon M., Martini F., Borghi A. (2020). Tissue cytokine/chemokine profile in vulvar lichen sclerosus: An observational study on keratinocyte and cultures. J. Dermatol. Sci..

[43] Mazziotta C., Badiale G., Cervellera C.F., Morciano G., Di Mauro G., Touzé A., Pinton P., Tognon M., Martini F., Rotondo J.C. (2024). All-trans retinoic acid exhibits anti-proliferative and differentiating activity in Merkel cell carcinoma cells via retinoid pathway modulation. J. Eur. Acad. Dermatol. Venereol..

[44] (2009). Biological Evaluation of Medical Devices—Part 5: Tests for In Vitro Cytotoxicity.

[45] Gruber S., Nickel A. (2023). Toxic or not toxic? The specifications of the standard ISO 10993-5 are not explicit enough to yield comparable results in the cytotoxicity assessment of an identical medical device. Front. Med. Technol..

[46] Leaper D.J., Schultz G., Carville K., Fletcher J., Swanson T., Drake R. (2012). Extending the TIME concept: What have we learned in the past 10 years?(*). Int. Wound J..

[47] Tottoli E.M., Dorati R., Genta I., Chiesa E., Pisani S., Conti B. (2020). Skin Wound Healing Process and New Emerging Technologies for Skin Wound Care and Regeneration. Pharmaceutics.

[48] Nuutila K., Eriksson E. (2021). Moist Wound Healing with Commonly Available Dressings. Adv. Wound Care.

[49] Das P., Manna S., Roy S., Nandi S.K., Basak P. (2023). Polymeric biomaterials-based tissue engineering for wound healing: A systemic review. Burns Trauma.

[50] Gallo A.L., Pollini M., Paladini F. (2018). A combined approach for the development of novel sutures with antibacterial and regenerative properties: The role of silver and silk sericin functionalization. J. Mater. Sci. Mater. Med..

[51] Rahman M., Dip T.M., Nur M.G., Padhye R., Houshyar S. (2024). Fabrication of Silk Fibroin-Derived Fibrous Scaffold for Biomedical Frontiers. Macromol. Mater. Eng..

[52] Babu P.J., Suamte L. (2024). Applications of silk-based biomaterials in biomedicine and biotechnology. Eng. Regen..

[53] Aldahish A., Shanmugasundaram N., Vasudevan R., Alqahtani T., Alqahtani S., Asiri A.M., Devanandan P., Thamaraikani T., Vellapandian C., Jayasankar N. (2024). Silk Fibroin Nanofibers: Advancements in Bioactive Dressings through Electrospinning Technology for Diabetic Wound Healing. Pharmaceuticals.

[54] El-Hamid M.I.A., Ibrahim D., Abdelfattah-Hassan A., Mohammed O.B., Pet I., Khalil S.S., El-Badry S.M., Metwally A.S., Azouz A.A., Elnegiry A.A. (2024). Silver nanoparticles loaded with pomegranate peel extract and hyaluronic acid mediate recovery of cutaneous wounds infected with Candida albicans. Front. Cell. Infect. Microbiol..

[55] Johnson K.E., Wilgus T.A. (2014). Vascular Endothelial Growth Factor and Angiogenesis in the Regulation of Cutaneous Wound Repair. Adv. Wound Care.

[56] Wilgus T.A. (2019). Vascular Endothelial Growth Factor and Cutaneous Scarring. Adv. Wound Care.

[57] Kim K.K., Sheppard D., Chapman H.A. (2018). TGF-β1 Signaling and Tissue Fibrosis. Cold Spring Harb. Perspect. Biol..

[58] Li C., Wang Q., Li J., Hu M., Shi S., Li Z., Wu G., Cui H., Li Y., Zhang Q. (2016). Silver nanoparticles/chitosan oligosaccharide/poly(vinyl alcohol) nanofiber promotes wound healing by activating TGFβ1/Smad signaling pathway. Int. J. Nanomed..

[59] Ortega M.M.C., Castillo J.M.Q., Del Castillo Castro T., Felix D.E.R., Ortega H.D.C.S., Manero O., Gastelum K.A.L., Chan L.H.C., Martinez D.H., Hernández J.A.T. (2024). Aloe vera mucilage loaded gelatin electrospun fibers contained in polylactic acid coaxial system and polylactic acid and poly(e-caprolactone) tri-layer membranes for tissue engineering. Biomed. Mater. Eng..

[60] Markandeywar T.S., Narang R.K. (2025). Collagen and chitosan-based biogenic sprayable gel of silver nanoparticle for advanced wound care. Naunyn. Schmiedebergs Arch. Pharmacol..

[61] Kolimi P., Narala S., Nyavanandi D., Youssef A.A.A., Dudhipala N. (2022). Innovative Treatment Strategies to Accelerate Wound Healing: Trajectory and Recent Advancements. Cells.

